# Regional-scale land-cover change during the 20th century and its consequences for biodiversity

**DOI:** 10.1007/s13280-014-0585-9

**Published:** 2015-01-09

**Authors:** Sara A. O. Cousins, Alistair G. Auffret, Jessica Lindgren, Louise Tränk

**Affiliations:** 1 Biogeography and Geomatics, Department of Physical Geography, Stockholm University, 106 91 Stockholm, Sweden; 2 County Administrative Board of Södermanland, Stora torget 13, 611 32 Nyköping, Sweden

**Keywords:** Habitat fragmentation, Historical ecology, Historical maps, Landscape change, Semi-natural grasslands, Species richness

## Abstract

Extensive changes in land cover during the 20th century are known to have had detrimental effects on biodiversity in rural landscapes, but the magnitude of change and their ecological effects are not well known on regional scales. We digitized historical maps from the beginning of the 20th century over a 1652 km^2^ study area in southeastern Sweden, comparing it to modern-day land cover with a focus on valuable habitat types. Semi-natural grassland cover decreased by over 96 % in the study area, being largely lost to afforestation and silviculture. Grasslands on finer soils were more likely to be converted into modern grassland or arable fields. However, in addition to remaining semi-natural grassland, today’s valuable deciduous forest and wetland habitats were mostly grazed grassland in 1900. An analysis of the landscape-level biodiversity revealed that plant species richness was generally more related to the modern landscape, with grazing management being a positive influence on species richness.

**Electronic supplementary material:**

The online version of this article (doi:10.1007/s13280-014-0585-9) contains supplementary material, which is available to authorized users.

## Introduction

Land-use change is thought to pose the most serious threat to biodiversity worldwide (Baillie et al. 2004). In Europe, the demand for food and fiber during the 20th century has resulted in widespread habitat loss, both through the intensification of agricultural land use and the abandonment of less-productive land. Many organisms are dependent on the agricultural landscape, and these changes have had negative effects on the biodiversity of plants, insects, and birds (Chamberlain et al. 2000; Krauss et al. 2010), in turn influencing the provision of a range of ecosystem services (Tscharntke et al. 2005). Many different habitat types in the rural landscape have been affected by intensification or abandonment. Productive grasslands and wetlands have been drained and converted to arable fields (Brinson and Malvárez 2002), and other low productive grassland areas have been fertilized or afforested (Ramankutty and Foley 1999; Poschlod and WallisDeVries 2002). Although the general trends of these past changes are quite well known, there are still not many accounts of how they manifest in the landscape at regional scales (but see Hooftman and Bullock 2012).

The overarching drivers behind land-use change have been technological advances, as well as political and socio-economic decisions or constraints. These are similar at regional, national, and international scales (Mattison and Norris 2005), but their effects on local and landscape scales are largely dependent on differences in soil fertility, topography or water availability (Lambin et al. 2001), and geographical location. For example, at the landscape scale, Cousins (2009a) found that areas with a larger proportion of clayey soils changed toward intensive crop production earlier, more than 100 years ago, than did areas with smaller proportions of clayey soil. On the other hand, arable fields and grasslands on more marginally productive soils have shown a tendency to be abandoned and afforested (Bender et al. 2005; Cousins 2009a; Hooftman and Bullock 2012). This means that not only have many important semi-natural habitats been lost, but the diversity of physical variation, wetness, and soil properties in remaining grasslands has also decreased.

Despite the magnitude of land-use change, plant species have often exhibited delayed responses to the associated habitat loss. Comparing present-day maps with historical maps and aerial photographs, researchers have regularly found that current plant species richness is more strongly related to past than present habitat area (Lindborg and Eriksson 2004; Helm et al. 2006; Vellend et al. 2006; Krauss et al. 2010). Many species, populations, or communities do not disappear directly following landscape or habitat change, but have been found to remain for at least 70 years in grasslands (Helm et al. 2006; Cousins 2009b; Plue and Cousins 2013), or even longer in ancient deciduous forests (Vellend et al. 2006). To understand the effects of landscape changes on species diversity and distributions, it is important to analyze what the landscape looked like further back in time. It has also been hypothesized that the magnitude of habitat loss can determine the presence or absence of such time lags, with a threshold suggested at around 10 % remaining habitat, after which biodiversity becomes synchronized with the reduced habitat cover (Cousins 2009b). Until now, the majority of studies evaluating how land-use and land-cover change impact biodiversity are generally carried out within one focal habitat type (Vellend et al. 2006; Krauss et al. 2010). Such studies often find that former land use still influences current diversity patterns, but this does not tell the whole story. At the wider scale, landscape complexity (hereafter landscape heterogeneity), is expected to play a primary role in determining biodiversity (Tscharntke et al. 2005). Furthermore, analyzing historical maps is time consuming and there are not always maps available covering larger regions. Older maps are usually very accurate for smaller areas, i.e., a few square kilometers, but due to small geometrical irregularities and inconsistent classifications, it can be difficult to compare them with present-day maps (Cousins 2001). However, to get a clear understanding of the effects of land-cover changes at a scale relevant for impacts on broad-scale biodiversity patterns, we need to get an understanding of common and everyday landscapes, away from the relatively well-preserved historical landscapes that are the focus for local conservation or restoration management.

Regional- or national-scale biodiversity atlases provide an excellent resource for analyzing broad-scale landscape effects on species patterns (Warren et al. 2001; Doxford and Freckleton 2012). Combining such data with good quality historical and present-day maps, we can expect to gain important insights into the effects of changes of both land-cover and landscape heterogeneity on biodiversity patterns today. It is clearly valuable not only to quantify land-use change and habitat loss, but also to try to understand the underlying reasons behind spatial patterns of change and their ecological consequences. To do this, we present a large-scale, high-resolution analysis of agricultural land cover between the beginning of the 20th century and today in a 1652 km^2^ area of southeastern Sweden. We relate land-cover trajectories to general soil types, and assess the consequences for present-day plant biodiversity. Specifically, we examine (1) the extent of habitat loss on the regional scale when both valuable and ‘uninteresting’ landscapes are included, focussing on different classes of semi-natural grassland, deciduous forest, and wetlands; (2) if land-cover change is related to particular soil type at the regional scale; (3) if present-day biodiversity and presence of red-listed species at landscape scales relate to broad habitat cover today and/or a century ago.

## Materials and methods

### Study area

**Fig. 1:**
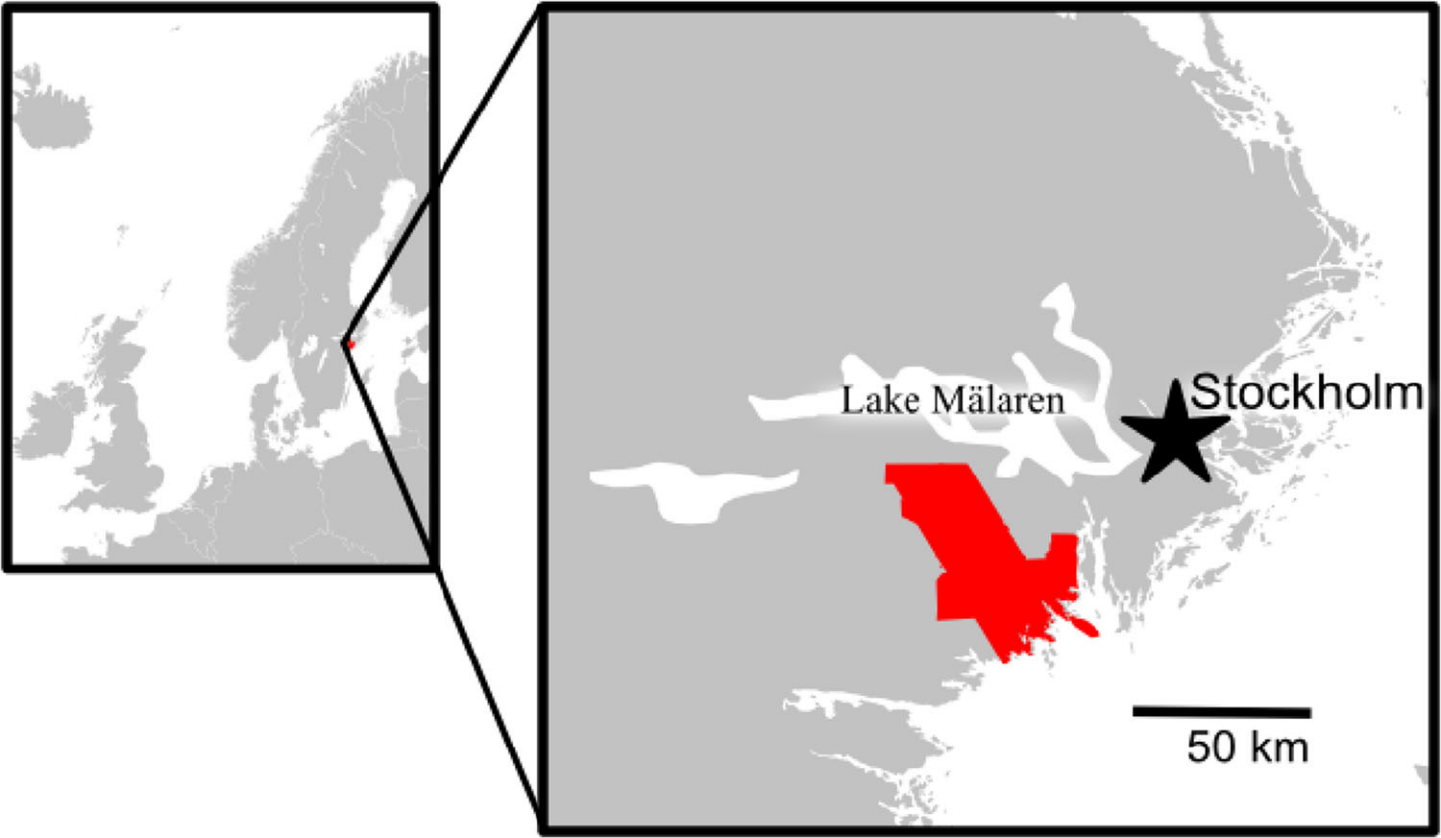
Map showing location of the study area used for a regional-scale analysis of land-cover change between 1900 and 2013

Our analysis is based on a study area covering 1652 km^2^ (midpoint 59°00′N, 17°11′E) in Södermanland (also known as Sörmland) county stretching from the Baltic coast in the south-east of the county towards the shores of lake Mälaren (Fig. 1). This region has been the basis of much grassland and historical ecological research during the past decade and is currently the subject of a transdisciplinary research program. The regional mean temperature for January is −4, and 16 °C for July, with a mean annual precipitation of 600 mm. The topography ranges from the Baltic Sea level to higher land with maximum altitude of 85 m. Forest, arable land, and lakes constitute the main part of the landscape today. The landscape has a long tradition of livestock grazing and haymaking.

### Map data

**Fig. 2:**
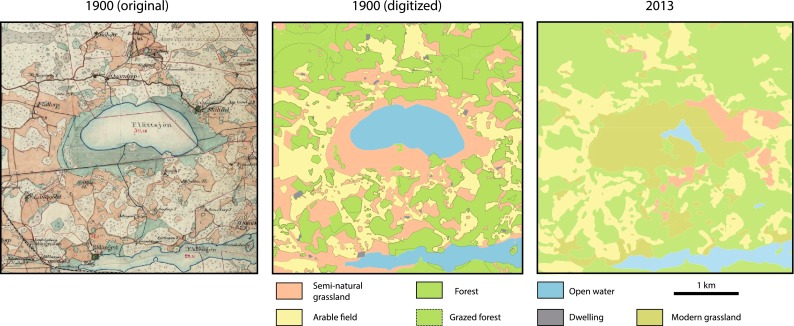
Cadastral map from 1900 over an area of a 1652 km^2^ study area compared with the corresponding digitized version and the map from 2013. Names, borders, roads, railway lines, and other linear objects were not included in the digitized version of the cadastral map. The colors in the original map have been changed in the digitized version to increase readability, thus the legend applies to the digitized maps only. *Dashed lines* indicate those areas we interpreted as wood pasture for analysis. The map from 2013 is based on the Swedish Lantmäteriet’s terrain map, overlaid with areas of semi-natural grassland from the Swedish government’s survey of semi-natural pastures and meadows

Old cadastral maps (in Swedish Häradskartan) at a scale of 1:50 000 from 1897–1901 (hereafter 1900) were digitized as a GIS vector layer (10.1007/s13280-014-0585-9). These old maps were economical maps showing land use, land cover, and ownership. The most important land uses at this time were crop fields and meadows, although forest, pastures, lakes, roads, dwellings, and other features were also mapped (Fig. 2). The rectification was carried out with a first polynomial transformation, and a total of 16 maps were used to cover the study area. The maps have a high resolution and accuracy (Jansson 1993), but there are small irregularities when transforming them, for example along the edges of each map when these are joined. Following rectification, the different land covers were manually digitized. To increase coverage, we also included previously digitized maps which were directly adjacent to the study area (e.g., Cousins and Vanhoenacker 2011). The digitized GIS layer included all the land-cover types (polygons) shown on the map, but linear and point features such as roads and place names were not digitized.

There was only one land-cover category for forest on the original map from 1900, meaning that no distinction was made between deciduous and coniferous forest, and grazed forest/wood pasture was not denoted. In fact, livestock grazed the outfield areas located outside the fenced-in village infields, a practice which took place from the time when fields became permanent during the Iron Age (Widgren 1983) until forest grazing was effectively banned in the 1940s. To incorporate wood pasture into the historical GIS layer, we classified all forests (excluding wetland areas) within 500 m of any dwelling as wood pasture. This is a conservative estimate, as the forests were intensively used for grazing, charcoal burning, and for collecting firewood. The resulting layer was the main historical data used for our analysis. Land-cover types were also grouped into broader categories, depending on the particular analysis being carried out (Table 1).

**Table 1:** Description of broad land-use categories and their subcategories from each time step. Subcategories from 1900 are from the Swedish cadastral map (Häradskartan), while the Swedish terrain map was used for the categories for 2013, supplemented with valuable semi-natural grasslands from the Swedish government’s survey of semi-natural pastures and meadows 2002–2004 (TUVA)

Broad land-use type	Subcategories 1900	Definition	Subcategories 2013	Definition
Arable field	Arable field	Area where crops were grown. Grazed post-harvest	Arable field	Area where crops are grown. Usually treated with chemical fertilizers and pesticides
Dwelling	Dwelling	Inhabited area	Dwelling	Inhabited area
Semi-natural grassland	Meadow (including wet meadows)	Land used to grow grass for livestock fodder	Meadow (TUVA)	Mowed historical grassland. Exist only for conservation purposes
	Pasture	Fenced areas for grazing	Pasture (TUVA)	Fenced areas of historical grassland used for intensive livestock grazing
	Wood pasture	Forest areas within 500 m of dwellings (own interpretation, see text)		
	Islet	Small areas within arable fields or meadows where cropping was not possible. Includes areas with coniferous, deciduous and no forest cover. Used for extra grazing and wood collection. Also includes small islands within lakes		
	Wetland (open)	Uncovered areas with a high soil moisture		
Forest	Wetland (deciduous and coniferous)	Wooded areas with a high soil moisture	Deciduous forest	Forest areas dominated by deciduous trees
	Forest	Wooded areas (excluding those within 500 m of a dwelling)	Coniferous and mixed forest	Forest areas dominated by coniferous species, occasionally mixed with deciduous species. Mainly silviculture
Modern grassland			Other open land	Usually grazed former arable fields and gardens
Open water	Open water	Inland lakes	Open water	Inland lakes

For present-day land cover, we used the 2013 terrain map (in Swedish Terrängkartan), which we simplified into fewer categories to become more comparable with the historical map (Table 1). To incorporate semi-natural grassland habitat into this map, we used the Swedish government’s survey of semi-natural pastures and meadows 2002–2004 (Updated 2012; TUVA database, www.sjv.se/tuva), replacing areas of the terrain map where currently managed semi-natural grasslands are located. Due to the marginal area of meadow today (0.4 km^2^; 1 % of total present-day semi-natural grassland), present-day semi-natural grassland was grouped as one category for all analyses. It must be noted that although most of the broad land-cover categories are shared between the two time steps, the character of forest, arable field, and semi-natural grasslands between 1900 and today are quite different (Table 1).

In addition to semi-natural grasslands, we also wanted to analyze the trajectories of the ecologically valuable habitats of wetlands and deciduous forest. Neither of these habitat types was particularly well mapped in the historical map, as all forests were lumped together as one category, and wetlands were not considered economically valuable and therefore not prioritized in the mapping process. Therefore, land-cover change in these habitats was analyzed backwards, i.e., we aimed to identify the historical land covers that became valuable habitats today. As much of the forest cover from the terrain map was classified as mixed deciduous and coniferous forest, we complemented the deciduous forest cover of the terrain map with the more detailed map layer of the Swedish national survey of broadleaf forest patches (in Swedish Ädellövskogsinventeringen). The wetland areas of the terrain map were complemented with a map layer of the Swedish national wetland inventory (in Swedish Våtmarksinventeringen). As both the inventories of the broadleaf forest and the wetlands sometimes overlapped the layer of the semi-natural grassland, they were not incorporated into the terrain map for any other analyses. A digital soil and bedrock database with a 5 m resolution (showing the soil type at 50 cm depth and at a scale of 1:50 000) from the Geological Survey of Sweden was acquired for analyzing land-cover change in relation to general soil properties.

### Land-cover change

Land-cover change was assessed by first calculating the areas of the broad land-cover types from the historical map, before overlaying each land-cover type separately onto the modified present-day terrain map and calculating the area of today’s land-cover categories within each historical land-cover type in question. Similarly, the historical land covers of present-day cover of wetland and deciduous forest were found by overlaying their current distributions over the historical map.

The patterns of grassland change in relation to soil types were evaluated by overlaying semi-natural grassland cover from both time steps onto the digital soil layer. For 1900, the soil distributions were calculated for total grassland area in the subcategories of grassland used primarily for haymaking (meadow and wet meadow) and grazing (pasture, wood pasture, and islets). Due to the uncertainties regarding the mapping of wetlands in 1900, open wetlands were not included in either of the above semi-natural grassland subcategories. All digitizing and analysis of land-cover change were carried out using ArcGIS 9 and 10 (ESRI, Redlands CA, USA).

### Biodiversity

To assess the influence of historical and present-day habitat availability on present-day biodiversity, we used the county plant atlas Sörmlands Flora (Rydberg and Wanntorp 2001), which is based on systematic inventories of the flora in the county of Södermanland carried out between 1980 and 1999. We extracted data for the plant taxa (hereafter species) present in the 5 × 5 km grid squares historically used in Sweden for landscape-level mapping. We split the study area according to these grid squares, removing those where the study area covered less than 90 % of the area of the square, resulting in 48 squares. For each square, the total species richness and the number of red-listed species from Sörmlands Flora were extracted from the Swedish Species Gateway (ArtPortalen, www.artportalen.se). A total of 1191 plant species were reported from the 48 squares, with a mean ± SD total of 552 ± 70 species and 12 ± 5 red-listed species per square. The area of different land-cover types were calculated in each square using PostGIS 1.5.3 (Holl and Plum 2009). As a measure of landscape heterogeneity, Shannon diversity of all broad land-cover types (Table 1) from both time steps was also calculated for each square.

The influence of habitat area and landscape heterogeneity across the two time steps on present-day plant species richness was analyzed using generalized linear models (GLM) with Poisson distributions. Our predictor variables were therefore: area of forest, area of semi-natural grassland and landscape heterogeneity (both time steps), and area of other open land (today only). Because of the collinearity of land cover and heterogeneity between years and within grid squares, the influence of all predictor variables on biodiversity was analyzed separately. Our statistical approach involved first creating a null model explaining grid-square level biodiversity, before adding one of our predictor variables to that null model. This new model was then compared with the null model using a Chi square test statistic to evaluate the significance of adding that predictor (De Frenne et al. 2011; Plue et al. 2013). This was carried out individually to predict the effect on both total species richness and the number of red-listed species per square. Statistical analyses were carried out using R 2.14.1 (R Development Core Team 2011; functions: *glm* and *anova*) with the additional library *vegan* (Oksanen et al. 2012; function: *diversity*).

## Results

### Land-cover change

**Fig. 3:**
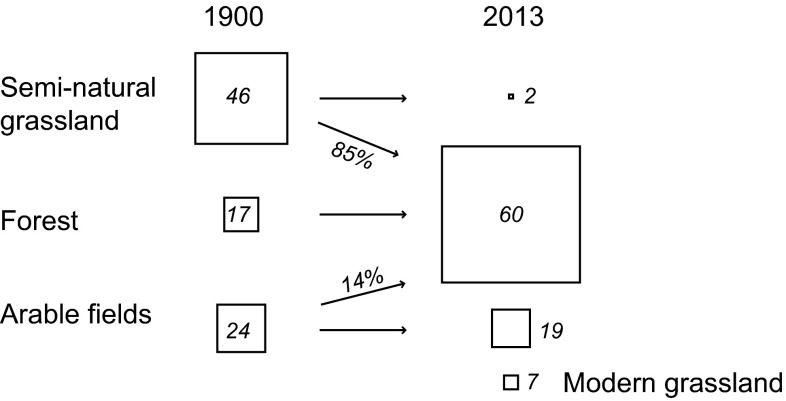
Major landscape transitions between 1900 and 2013 in a 1652 km^2^ transect in southeastern Sweden. *Boxes* are proportional to the change where the *italic number* gives the percentage of total land cover in the study area. *Arrows* show the dominant transitions to another land cover. Modern grassland derives from different historical land-cover categories but were primarily semi-natural grassland that have been used as arable fields between 1900 and today, or semi-natural grasslands that have been improved with fertilizers. Open water and dwellings are not included in the figure

The area of semi-natural grassland shrank dramatically to less than 4% of its previous cover, because of increasing silviculture (Fig. 3). Arable field also declined, either becoming forest or modern grassland. Modern grassland, which did not exist in 1900, covers more than three times the present-day semi-natural grassland area.

**Fig. 4:**
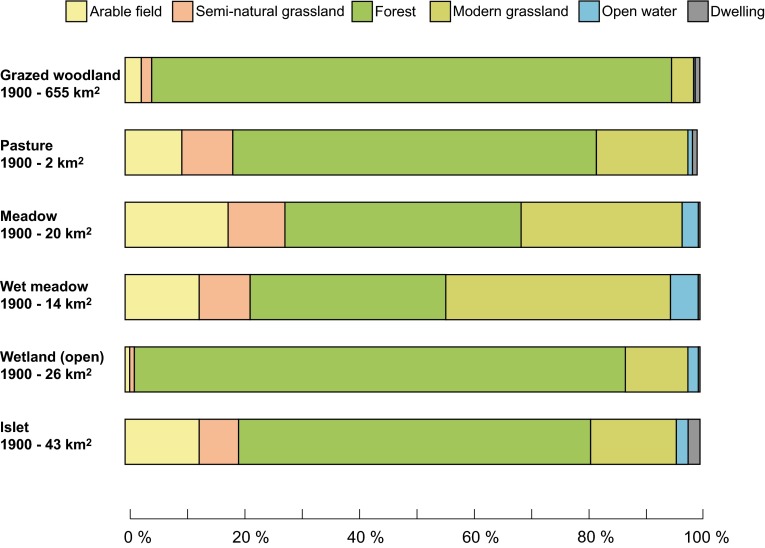
Land-cover trajectories for the different subcategories of semi-natural grassland to broad current land-cover types between 1900 and 2013 over a 1652 km^2^ study area in southeastern Sweden. Note that the area of dedicated pasture was relatively small in 1900, compared to islets and wood pasture where the majority of grazing took place

**Fig. 5:**
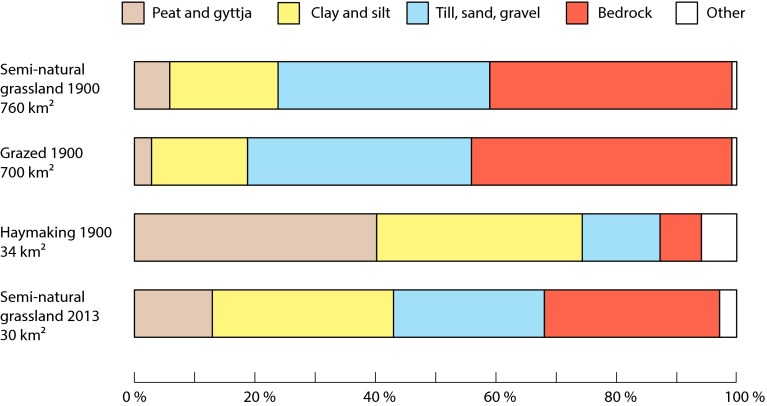
General soil types underlying different categories of semi-natural grassland in 1900 and 2013 over a 1652 km^2^ study area in southeastern Sweden. Grazed grassland 1900 includes pasture, wood pasture, and islets, while grassland tended for haymaking included meadow and wet meadow. *Bar colors* are based on those used by the Geological Survey of Sweden

The land-cover trajectories of semi-natural grassland between 1900 and today were related to both the subcategory of grassland and soil properties. The vast majority of grazed pasture became forest, while meadows were more likely to become arable field or modern grassland (Fig. 4). Islets, which were grazed in 1900, are now either isolated in arable fields or have been incorporated into the land cover surrounding these small habitats. With regards to soil, the distribution of semi-natural grasslands as a whole did not appear to change dramatically between 1900 and 2013 (Fig. 5). Broad soil types were divided quite evenly between bedrock, finer sediments, and the larger till and sand fractions, with a smaller area of organic peat and gyttja. However, dividing the semi-natural grasslands into those primarily used for grazing and haymaking showed that haymaking usually occurred on finer soils, whereas grazing took place on coarse-grained soils.

**Fig. 6:**
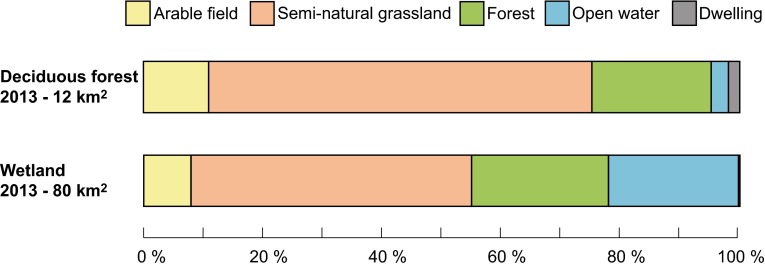
Broad land cover in 1900 for the present-day (2013) distributions of deciduous forest and wetland over a 1652 km^2^ study area in southeastern Sweden

The areas of present-day valuable wetlands and deciduous forest within the study area were to a large extent, 50 and 70 % respectively, managed as semi-natural grassland in 1900 (Fig. 6). The present-day wetlands were used as meadows in the past and the deciduous forests for grazing. Around 30% of both habitat types were either forest or managed as arable field in the past, while many wetland areas were mapped as open water a century ago (for example the lake in Fig. 2).

### Biodiversity

Both landscape heterogeneity and the cover of forest and grassland were related to the total plant species richness and the number of red-listed species within the 5 × 5 km grid squares covered by the study area. Present-day landscape heterogeneity and the current area of semi-natural grassland and other open land were positively related to both total species number and the number of red-listed species (Table 2). Forest cover was negatively related to total plant species number and number of red-listed species at both time steps, but historical cover of semi-natural grassland had no effect. Landscape heterogeneity in 1900 was positively related to total diversity today, while this was not true for red-listed species.

**Table 2:** The influence of past and present habitat area and heterogeneity (Shannon diversity) on the total number of species and red-listed species within a grid of 48 5 × 5 km squares in southeastern Sweden based on generalized linear models and subsequent Chi square test statistics

	Total species richness			Red-listed species		
	Effect	Chi square	*P*	Effect	Chi square	*P*
1900						
Forest	−	101	<0.001	−	18.29	<0.001
Semi-natural grassland		3.31	0.07		0.97	0.32
Landscape heterogeneity	+	13.25	<0.001		2.01	0.16
2013						
Forest	−	115	<0.001	−	21.42	<0.001
Other open land	+	63.38	<0.001	+	11.63	<0.001
Semi-natural grassland	+	76.7	<0.001	+	17.45	<0.001
Landscape heterogeneity	+	150.45	<0.001	+	27.38	<0.001

## Discussion

### Land-cover change

Ninety-six percent of semi-natural grassland area has disappeared from the study area during the 20th century, mostly to become forest. This is more than has previously be found in landscapes in and around the transect area (Cousins et al. 2007; Cousins and Eriksson 2008; Cousins 2009a), indicating that habitat loss might be more severe than expected from studies that have been more focused on areas close to villages or within the infield area.

Despite these large losses, the patterns of soil distribution below semi-natural grasslands did not show much change between 1900 and today. This is probably because semi-natural grasslands in both time steps were dominated (~ 99%) by grazing. Haymaking was concentrated on finer soils, which were largely converted to arable fields or modern grasslands (Fig. 5), while grazing lands on less-productive soil were largely abandoned. The relatively high proportion of semi-natural grassland today located on highly productive clayey soils, instead of less-productive soils, are more likely due to the generous classification in the national survey of valuable grasslands. In the classification of semi-natural grasslands today it often includes both historical grassland on coarser, less-productive soils and former arable fields on clayey soils. Although these results show that diversity of grasslands with regards to soil has probably not changed on the regional scale during the past 100 years, the sheer magnitude of the destruction of grasslands means that those historical grasslands located on finer soils have shrunk to an almost insignificant size—0.4 km^2^ compared to 20 km^2^ in 1900. Furthermore, many meadows in this landscape disappeared prior to 1900 during the agrarian revolution (Cousins 2009a; Eriksson and Cousins 2014).

Although our analyses concentrated on the absolute and relative changes in different land-cover categories during the 20th century, other changes in the landscape can also affect ecological processes in the landscape. For example, connectivity between habitats (Hooftman and Bullock 2012; Auffret et al. 2015) or the variation of management intensity within each land-cover categories are also very relevant. Furthermore, more than 100 years is a long time period for analyzing changes in land cover, and many additional changes will have happened during the 20th century. The largest extent of crop fields in this region in Sweden was in the 1920s (Mattsson 1985), when all plowable soils were cultivated. For the landscapes within the study area this would have meant that most meadows and many wetlands mapped in 1900 had by that time been drained and cultivated. Later, these are likely to have changed again to become grazed modern grasslands, which hold fewer species compared to semi-natural grasslands (Auffret and Cousins 2011; Marteinsdottir and Eriksson 2014), but we can see positive effects of modern grasslands within the broad-scale analyses of species richness.

In addition to remaining semi-natural grasslands, deciduous forests and wetlands are also of high ecological value in the rural landscape (Brinson and Malvárez 2002; Gilliam 2007). As they were not of interest economically in 1900 and therefore not accurately mapped, we have used present-day surveys of deciduous forest and wetlands to reveal which land-cover categories they were in the past. Both were primarily used as grasslands in 1900, and could therefore today potentially represent successional stages between grassland and future climax communities. A range of wetland types were mapped in the historical map, both open or with a canopy cover (deciduous or coniferous). However, as these were not mapped with any great accuracy it was not possible to analyze how they have changed. On the other hand, wet meadow and meadow (usually moist) were economically valuable in 1900 and thus mapped, of which both have more or less completely disappeared today.

### Biodiversity

Biodiversity and the presence of red-listed species are apparently in synchrony with land-cover patterns in the modern-day landscape at the broad scale. This finding is contrary to many studies based on agricultural landscapes, which often report that plant diversity is more related to historical habitat cover and land-use configurations (Lindborg and Eriksson 2004; Helm et al. 2006; Krauss et al. 2010). However, by being focused on one habitat of interest or at a fine-scale landscape study, such investigations are necessarily conducted in landscapes with a certain minimum degree of focal habitat still intact. Here we included a wide variety of landscapes, and in accordance with the relatively large magnitude of land-cover change, the effects on biodiversity also appear to exceed those reported from smaller scale investigations. Even though modern grasslands were also found to be positively related to biodiversity, the huge decline in semi-natural grassland habitat has probably exceeded any extinction threshold which might exist for biodiversity to relate to historical habitat cover (see Fahrig 2003; Cousins 2009b).

The negative relationship between biodiversity and forest cover at both time steps appears quite logical when viewed in the context of our results relating to land cover. What was forest in 1900 is to a large extent still forest today (Figs. 2, 3). Much of today’s forest is managed, and is therefore expected to host a low plant species diversity compared to other types of habitat (Hartley 2002). Deciduous forest areas, which are thought to be of high value to biodiversity (Gilliam 2007), were to a large degree actually managed semi-natural grasslands in 1900 (Fig. 6). Today, they only occupy less than 0.01 % of the total area, and are so small that any positive effects on biodiversity would be effectively swallowed up by the negative effects of the silviculture, which dominates their shared broad habitat category.

Landscape heterogeneity at both time steps was found to be positively related to biodiversity. This lends further support to the value of considering whole landscapes as opposed to focal habitats for assessing land-cover change and its ecological effects. Tscharntke et al. (2005) argue that land-use heterogeneity enhances diversity at landscape scales, and we also find that historical heterogeneity can retain an influence over present-day biodiversity patterns. Although the positive effect of grassland may disappear after a certain degree of habitat loss, the net effect of a diversity of different land-cover types within a historical landscape still appears to influence biodiversity today. However, despite otherwise showing the same effects as total species richness, the number of red-listed species within a grid square was not related to past landscape heterogeneity. Other studies considering both total species richness and habitat specialists have found that they can exhibit different responses to land-use change (Cousins and Vanhoenacker 2011; Piqueray et al. 2011). In our case it appears that red-listed species might be the first to react to any changes in landscape, but without any intermediate time steps, it is not possible to know if this was also the case with the relationship between total diversity and area of semi-natural grassland. Our understanding of the changing relationships between biodiversity and land cover would also have been improved with historical biodiversity data corresponding to the time the maps were made, though this is rare (but see Jiang et al. 2013).

Finally, the potential pitfalls of the plant atlas data, such as different individuals carrying out the inventories and the long time span of the data collection, should be noted. However, we believe that the benefits of using biodiversity at the whole landscape scale rather than in a few choice habitats far outweighs these concerns, and the systematic inventory used for the flora (Rydberg and Wanntorp 2001) means that the issue of false absences which can affect other data drawn from large-scale biodiversity observation data sets (Phillips et al. 2009) are far less relevant here. Finally, the broad 5 × 5 km scale of the biodiversity data in contrast to the fine-scale land-cover data means that some within-square variation in land-cover-related biodiversity will be hidden. For example, this would mask the contributions of any habitats of exceptional diversity to the landscape-level biodiversity, although the identification of such habitats would be of value for conservation management. However, the fact that the presence of grassland habitats and increased landscape heterogeneity were related to increased species richness indicates that vegetation data at this scale can still be useful for larger scale analyses of land cover and biodiversity.

## Conclusion

We have presented the first digitization and analysis of century old historical maps for such a large region. Our high-resolution analysis of land-cover change during the 20th century revealed that the magnitude of habitat destruction can be more severe when including a larger section of the landscape compared to previous landscape-scale studies. This is important not only because of the direct effects of habitat loss on biodiversity, but also through the consequences that land-use change has on services such as natural resource availability and air and water quality (Foley et al. 2005). Additionally, the increased habitat change will also have implications for the ecological responses of organisms to climatic changes by altering the dynamics and interactions of ecological populations and communities (Opdam and Wascher 2004; Elmhagen et al. 2015, Navarro-Cano et al. 2015; Strandmark et al. 2015). Therefore, we believe that large, high-resolution studies of land-cover change are of great value for gaging the true extent of habitat destruction, and can be an important prerequisite for understanding and managing the rural landscape and its biodiversity.

## Electronic supplementary material

Below is the link to the electronic supplementary material.

Supplementary material 1 (PDF 47 kb)

Supplementary material 2 (SHX 208 kb)

Supplementary material 3 (DBF 883 kb)

Supplementary material 4 (PRJ 1 kb)

Supplementary material 5 (QPJ 1 kb)

Supplementary material 6 (SHP 34,547 kb)
