# Supplementary material for: Regional-scale land-cover change during the 20th century and its consequences for biodiversity
Source: Ambio. 2015 Jan 9;44(Suppl 1):17–27. doi: 10.1007/s13280-014-0585-9 (PMC4288995; doi:10.1007/s13280-014-0585-9)
Supplement: Supplementary file 1 — Supplementary material 1 (PDF 47 kb) [file 13280_2014_585_MOESM1_ESM.pdf]

AMBIO

Electronic Supplementary Material

This supplementary material has not been peer reviewed.

Title: **Regional-scale land-cover change during the 20th century and its consequences for biodiversity**

Authors: Cousins, S.A.O., A.G. Auffret, J. Lindgren, and L. Tränk

Geographical Information System (GIS) vector layer over a 1652 km2 transect in southeastern Sweden showing land cover in 1900. The vector layer includes five files that can be uploaded and displayed in a GIS program; dbf; prj; qpj; shp; shx.

Background map was the Swedish Lantmäteriet's Häradskartan, digitized using ArcGIS 9.3 (ESRI, Redlands CA, USA).

The file contains the following land-cover categories (column: LANDCOVER) based on map colors, symbols and accompanying text. See Table 1 in article for descriptions.

Arable field

Dwelling

Forest

Islet

Meadow

Open water

Pasture

Wet meadow

Wetland, coniferous

Wetland, deciduous

Wetland, open

Coordinate system SWEREF99 TM (ESPG: 3006).
